# Gabapentin Modulates HCN4 Channel Voltage-Dependence

**DOI:** 10.3389/fphar.2017.00554

**Published:** 2017-08-21

**Authors:** Han-Shen Tae, Kelly M. Smith, A. Marie Phillips, Kieran A. Boyle, Melody Li, Ian C. Forster, Robert J. Hatch, Robert Richardson, David I. Hughes, Brett A. Graham, Steven Petrou, Christopher A. Reid

**Affiliations:** ^1^Florey Institute of Neuroscience and Mental Health, The University of Melbourne, Parkville VIC, Australia; ^2^School of Biomedical Sciences and Pharmacy, University of Newcastle, Callaghan NSW, Australia; ^3^Hunter Medical Research Institute, New Lambton Heights NSW, Australia; ^4^School of BioSciences, The University of Melbourne, Parkville VIC, Australia; ^5^Institute of Neuroscience and Psychology, University of Glasgow Glasgow, United Kingdom

**Keywords:** HCN4, gabapentin, pain, epilepsy, spinal cord

## Abstract

Gabapentin (GBP) is widely used to treat epilepsy and neuropathic pain. There is evidence that GBP can act on hyperpolarization-activated cation (HCN) channel-mediated *I*_h_ in brain slice experiments. However, evidence showing that GBP directly modulates HCN channels is lacking. The effect of GBP was tested using two-electrode voltage clamp recordings from human HCN1, HCN2, and HCN4 channels expressed in *Xenopus* oocytes. Whole-cell recordings were also made from mouse spinal cord slices targeting either parvalbumin positive (PV^+^) or calretinin positive (CR^+^) inhibitory neurons. The effect of GBP on *I*_h_ was measured in each inhibitory neuron population. HCN4 expression was assessed in the spinal cord using immunohistochemistry. When applied to HCN4 channels, GBP (100 μM) caused a hyperpolarizing shift in the voltage of half activation (*V*_1/2_) thereby reducing the currents. Gabapentin had no impact on the *V*_1/2_ of HCN1 or HCN2 channels. There was a robust increase in the time to half activation for HCN4 channels with only a small increase noted for HCN1 channels. Gabapentin also caused a hyperpolarizing shift in the *V*_1/2_ of *I*_h_ measured from HCN4-expressing PV^+^ inhibitory neurons in the spinal dorsal horn. Gabapentin had minimal effect on *I*_h_ recorded from CR^+^ neurons. Consistent with this, immunohistochemical analysis revealed that the majority of CR^+^ inhibitory neurons do not express somatic HCN4 channels. In conclusion, GBP reduces HCN4 channel-mediated currents through a hyperpolarized shift in the *V*_1/2_. The HCN channel subtype selectivity of GBP provides a unique tool for investigating HCN4 channel function in the central nervous system. The HCN4 channel is a candidate molecular target for the acute analgesic and anticonvulsant actions of GBP.

## Introduction

(GBP) was synthesized as a rigid lipophilic analog of gamma-aminobutyric acid (GABA) with the intention of mimicking inhibitory neurotransmission. Although GBP was first approved for the treatment of epilepsy it is now more commonly used as a therapy in neuropathic pain. In addition, it is often used off-label to treat a number of medical conditions including anxiety, alcohol and drug addiction, and bi-polar disorders (Mack, 2003). Despite the rational design, there is no current evidence that GBP directly modulates the GABAergic system (Taylor et al., 2007). Instead, the widely accepted mode-of-action of GBP is that it interacts with the α2δ protein, an accessory subunit of voltage-gated calcium (Ca^2+^) channels responsible for enhancing channel trafficking to the plasma membrane as well as influencing their biophysical properties (Dolphin, 2016). Gabapentin interaction with the α2δ subunit is thought to lead to reduced voltage-gated Ca^2+^ channel trafficking to the membrane surface of glutamatergic synapses, reducing excitatory neurotransmitter release, and consequently excitability (Fink et al., 2002). Gabapentin has also been shown to acutely block voltage-dependent Ca^2+^ channels (Stefani et al., 1998; Sutton et al., 2002) and putatively interacts with several molecular targets including *N*-methyl-D-aspartic acid (NMDA) and α-amino-3-hydroxy-5-methyl-4-isoxazolepropionic acid (AMPA) receptors, and K_ATP_ channels (Cheng and Chiou, 2006).

In this study, we investigate hyperpolarization-activated cation (HCN) channels as a potential target of GBP. Hyperpolarization-activated cation channels are encoded by four genes, *HCN1*–*HCN4* and are expressed in brain and cardiac tissue (Santoro and Tibbs, 1999). Hyperpolarization-activated cation channels carry a non-selective cationic conductance (*I*_h_) that regulates neuronal excitability by modulating resting membrane potential, input resistance, and synaptic integration (He et al., 2014). Several studies have mechanistically linked changes in the function and expression of various HCN channels to epilepsy and pain, making them good molecular targets in both neurological conditions (Reid et al., 2012; Benarroch, 2013; DiFrancesco and DiFrancesco, 2015; Tibbs et al., 2016). Furthermore, studies using hippocampal slices show that GBP increases *I*_h_ in both pyramidal neurons (Surges et al., 2003) and inhibitory interneurons in CA1 (Peng et al., 2011). Although these studies imply a GBP effect on *I*_h_ currents, whether GBP acts directly on HCN channels in isolation is not clear. Here, we tested the effect of GBP on heterologously expressed human HCN1, HCN2, and HCN4 channels. Our results show that GBP shifts the voltage dependence of activation of HCN4-mediated currents. This effect was also true for native HCN4-mediated currents in the mouse central nervous system, with GBP shifting the voltage dependence of activation of *I*_h_ in parvalbumin (PV)-expressing inhibitory neurons in the spinal cord that are known to express HCN4 channels (Hughes et al., 2013), but not in inhibitory calretinin (CR)-expressing interneurons that we show lack HCN4. These results highlight HCN4 channels as a potential pharmacological target of GBP. They also suggest that drugs with absolute HCN4 subunit selectivity may be possible.

## Materials and Methods

### Animal Ethics

Procedures using animals in this study were conducted in accordance with the Prevention of Cruelty to Animals Act 1986, under the guidelines of the NHMRC Code of Practice for the Care and Use of Animals for Experimental Purposes in Australia and were approved by the Florey Neuroscience Institute and the Newcastle University Animal Ethics Committees. Experiments completed in the United Kingdom were approved by the University of Glasgow Animal Ethics Committee.

### HCN cRNA *In Vitro* Transcription

The human(h) HCN1 transcript was cloned by Dr. Ludwig and Dr. Stieber. The pcDNA3 hHCN1, and pSGEM hHCN4 clones came from on-going collaborations with Dr. Lerche. The pcDNA3 hHCN2 clone was obtained from Dr. Biel (Ludwig et al., 1999). The HCN1 and HCN2 inserts were sub-cloned into the oocyte expression vector, pGEMHE-MCS and confirmed by DNA sequencing (Department of Pathology, The University of Melbourne, Australia). *In vitro* synthesis of cRNA was performed using the mMessage mMachine^®^ T7 transcription kit (Ambion, Foster City, CA, United States).

### HCN Two-Electrode Voltage Clamp Experiments in Oocytes

Oocytes from *Xenopus laevis* were prepared as previously described (Dibbens et al., 2010). Briefly, 50 nl of cRNA-encoding human HCN subunits (12.5 ng/μl; concentration confirmed spectrophotometrically and by gel analysis) was injected into stage V–VI oocytes (Dumont’s classification; 1200–1300 μm in diameter) using the Roboocyte (Multi Channel Systems, Reutlingen, Germany) and the oocytes were incubated for 2 days at 15°C prior to experimentation. For heteromeric HCN combinations, equal concentrations of each subunit were premixed and injected into the oocytes.

Oocytes were perfused with a bath solution comprising of (in millimolar) 96 KCl, 2 NaCl, 2 MgCl_2_, and 10 HEPES (pH 7.5 using KOH). Following baseline readings, oocytes were perfused with bath solution only (vehicle control) or bath solution containing GBP (50 or 100 μM) for 1 min before recording. Voltage clamp experiments were performed using Roboocyte and Roboocyte2 systems at room temperature (22–25°C). Oocytes were impaled with two glass electrodes containing 1.5 M potassium acetate and 0.5 M KCl, clamped at a holding potential of -30 mV. Incremental 5 mV voltage steps (-95 to -30 mV for hHCN1, -100 to -45 mV for hHCN2, -110 to -45 mV for hHCN4, and -110 to -30 mV for heteromeric hHCN2+4) were applied for 15 s, followed by a step to -100 mV for 2 s. Comparisons of *I*_h_ under each condition were made using normalized G–V relationships, where peak tail current amplitudes recorded during the -100 mV test pulse were divided by the largest peak tail current, plotted against prepulse voltage and fit with a Boltzmann function (GraphPad Prism 7; GraphPad Software, La Jolla, CA, United States). The time to half the peak steady-state amplitude was used as a measure of the rate of HCN channel activation at various voltages.

### Mouse Spinal Cord Slice Electrophysiology

Spinal cord slices were prepared as previously described (Graham et al., 2003). Transgenic mice (age 4–8 months) expressing enhanced green fluorescent protein (eGFP) in CR (*n* = 6 mice) (Caputi et al., 2009) and PV (*n* = 7 mice) (Meyer et al., 2002) positive neurons, referred to hereafter as CReGFP and PVeGFP, respectively, were anesthetized with ketamine (intraperitoneally at 100 mg kg^-1^) and decapitated. The spinal cord was rapidly dissected free of the vertebral column and placed in ice-cold sucrose substituted artificial cerebrospinal fluid (aCSF) containing (in millimolar): 250 sucrose, 25 NaHCO_2_, 10 glucose, 2.5 KCl, 1 NaH_2_PO_4_, 1 MgCl_2_, and 2.5 CaCl_2_ for cutting. Parasagittal spinal cord slices (L1–L5; 200 μm thick) were prepared using a vibratome (Camden 7000 smz; Campden Instruments, Loughborough, Leicestershire, United Kingdom) and transferred to an incubation chamber containing aCSF (118 mM NaCl substituted for sucrose) saturated with carbogen gas (consisting of 95% O_2_ and 5% CO_2_). Slices were allowed to equilibrate for at least 1 h at room temperature prior to recording. Slices were transferred to a recording chamber and constantly superfused with carbogen-bubbled aCSF at a flow rate of ∼2 ml/min. Tetrodotoxin (TTX) (1 μM) was included in the aCSF to prevent unclamped sodium spikes contaminating traces. All recordings were made at room temperature from visualized inhibitory CReGFP^+^ or PVeGFP^+^ neurons in the spinal dorsal horn under fluorescence using a 488 nm excitation and 508 nm emission filter set. Importantly, CReGFP^+^ neurons include inhibitory and excitatory populations in the spinal dorsal horn; however, the inhibitory subtype is easily distinguished in spinal slices by extensive dendritic arbors in the rostrocaudal plane (Smith et al., 2015). In contrast, PVeGFP^+^ dorsal horn neurons are predominantly inhibitory (Hughes et al., 2012). Patch pipettes (6–8 MΩ) were filled with internal solution containing (in millimolar): 135 K-gluconate, 6 NaCl, 2 MgCl_2_, 10 HEPES, 0.1 EGTA, 2 MgATP, 0.3 NaGTP, and 0.2% Neurobiotin (pH 7.3 with KOH).

*I*_h_ currents were studied under voltage-clamp from a holding potential of -50 mV. The I–V relationship for *I*_h_ was determined by delivering incrementing prepulses in 5 mV from -100 to -50 mV (2 s duration) followed by a 1 s test pulse at -100 mV (10 s sweep intervals). These data were collected at both baseline and following bath application of GBP (100 μM) for 3–5 min. All data were acquired using a Multiclamp 700B amplifier (Molecular Devices, Sunnyvale, CA, United States), digitized online (sampled at a 10–20 kHz and filtered at 5 kHz), and stored using Axograph X software (Axograph Scientific, Sydney, Australia). Recordings were only included for analysis if the series resistance was <30 MΩ, filtered at 5 kHz and did not change more than 10% throughout the recording. Data were analyzed offline using Axograph software. Normalized G–V relationships were constructed using the tail currents recorded during each -100 mV test pulse response, divided by the largest peak tail current, plotted against prepulse voltage and fit with a Boltzmann curve to determine *V*_1/2_ and slope.

### Immunohistochemistry

A total of three adult male C57Bl6 mice (20–22 g) were deeply anesthetized with pentabarbitone and perfused transcardially with 4% depolymerized formaldehyde. The lumbar spinal cord was removed and post-fixed in the same solution for an additional 2 h. Spinal cord sections (60 μm thick, transverse planes) were cut on a vibratome and subsequently incubated in 50% ethanol for 30 min to enhance antibody penetration.

Free-floating sections were incubated in goat anti-CR (1:1000; SWANT, Bellinoza, Switzerland; Schiffmann et al., 1999), rabbit anti-Pax2 (1:1000; Invitrogen, Paisley, United Kingdom; Dressler and Douglass, 1992), and mouse anti-HCN4 (1:500; UC Davis/NIH NeuroMab Facility, Davis, CA, United States; Giesbrecht et al., 2010; Khurana et al., 2012). Hyperpolarization-activated cation-4 labeling was visualized using a tetramethylrhodamine kit (PerkinElmer Life Sciences, Boston, MA, United States) for tyramide signal amplification as described previously (Hughes et al., 2012), whereas immunolabeling for CR and Pax2 was visualized using species-specific secondary antibodies conjugated to Alexa 647 and Pacific Blue, respectively. All primary and secondary antibody cocktails were made up in 0.3 M phosphate-buffered saline with 0.3% Triton X-100. Sections were incubated in primary antibodies for 72 h and in secondary antibodies for 12–18 h at 4°C.

Representative sections from each animal were scanned on a confocal microscope (Zeiss LSM710, Hemel Hempstead, United Kingdom). Mosaics of overlapping confocal image stacks centered on lamina II were collected using a 40× objective (0.9 digital zoom, 1 μm z-separation) to determine the expression of HCN4 immunolabeling in Pax2-expressing CR cells. These mosaics were analyzed off-line using “Neurolucida for Confocal” software (MicroBrightField, Colchester, VT, United States) to determine the expression pattern of HCN4 immunolabeling in the cell bodies of Pax2-expressing CR cells. Calretinin cells that expressed Pax2 were initially identified, before revealing the HCN4 channel to determine the presence of immunolabeling, as described previously (Hughes et al., 2013). Cells were defined as expressing HCN4 if immunolabeling was confined to the cell membrane of individual cells, determined by following individual cells throughout the z-series of the confocal stack.

### Drugs and Statistics

Tetrodotoxin was purchased from Alomone Laboratories (Jerusalem, Israel), and GBP was purchased from Tocris Bioscience (Bristol, United Kingdom). Drugs were stored at 1000× final concentration and diluted in oocyte bath solution or aCSF prior to application. All data were pooled and represent means ± standard error of the mean (SEM). Data sets were compared using Student’s *t*-test unless stated (GraphPad Prism 7; GraphPad Software, La Jolla, CA, United States). An *F* test was applied to determine equality of variance before parametric statistical analysis was applied (GraphPad Prism). Differences were deemed statistically significant when *p* < 0.05.

## Results

### GBP Changes the Biophysical Properties of HCN4 Channels

**FIGURE 1:**
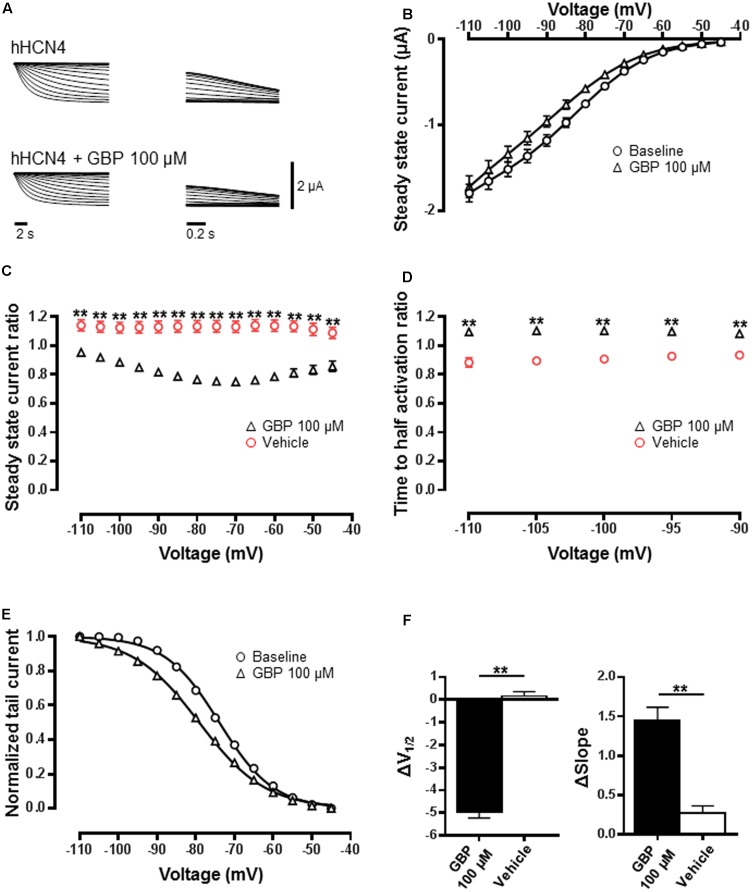
HCN4 channel function is reduced by GBP. **(A)** Raw HCN4 channel-mediated steady-state (left) and tail currents (right) before and after GBP. **(B)** Average steady-state current before and after GBP. **(C)** Average 100 μM GBP to vehicle steady-state current ratio at various voltages. **(D)** Average 100 μM GBP to vehicle time to half-activation ratio at various voltages. **(E)** Normalized conductance–voltage relationship constructed from tail currents. **(F)** Average shift (Δ) in the voltage of half-activation and slope of HCN4 channel by 100 μM GBP and vehicle control, relative to baseline measurements. ^∗∗^*p* < 0.0001.

Gabapentin (100 μM) had a visual impact on several HCN4 channel biophysical properties (**Figures 1A,B**; *n* = 50). As contemporaneous vehicle controls revealed changes in several of the HCN4 biophysical properties statistical analysis was made by comparing the relative change caused by GBP (100 μM) to the relative change observed in vehicle control. Gabapentin caused a significant reduction in steady-state current across all voltages relative to vehicle control (*n* = 23) (**Figure 1C**; *p* < 0.0001). There was also a significant slowing in the time to activation across a range of voltages (**Figure 1D**; *p* < 0.0001). The normalized conductance–voltage relationship of HCN4 currents was left-shifted by ∼5 mV after GBP (100 μM) application (**Figures 1E,F**). Half activation voltage (*V*_1/2_), estimated by a Boltzmann fit was significantly different relative to vehicle control (**Figure 1F**; *p* < 0.0001). A significant difference in the slope of the curve between baseline and GBP was also observed (**Figure 1F**; *p* < 0.0001). GBP (50 μM) had no effect on *V*_1/2_ (control *V*_1/2_ = 81.2 ± 2.0 mV vs. GBP 50 μM *V*_1/2_ = 81.4 ± 1.4 mV, *n* = 7, *p* = 0.9) or slope (control slope = 6.3 ± 0.2 vs. GBP 50 μM slope = 7.3 ± 0.5 mV, *n* = 7, *p* = 0.1).

### GBP Effect on HCN1 and HCN2 Channels

**FIGURE 2:**
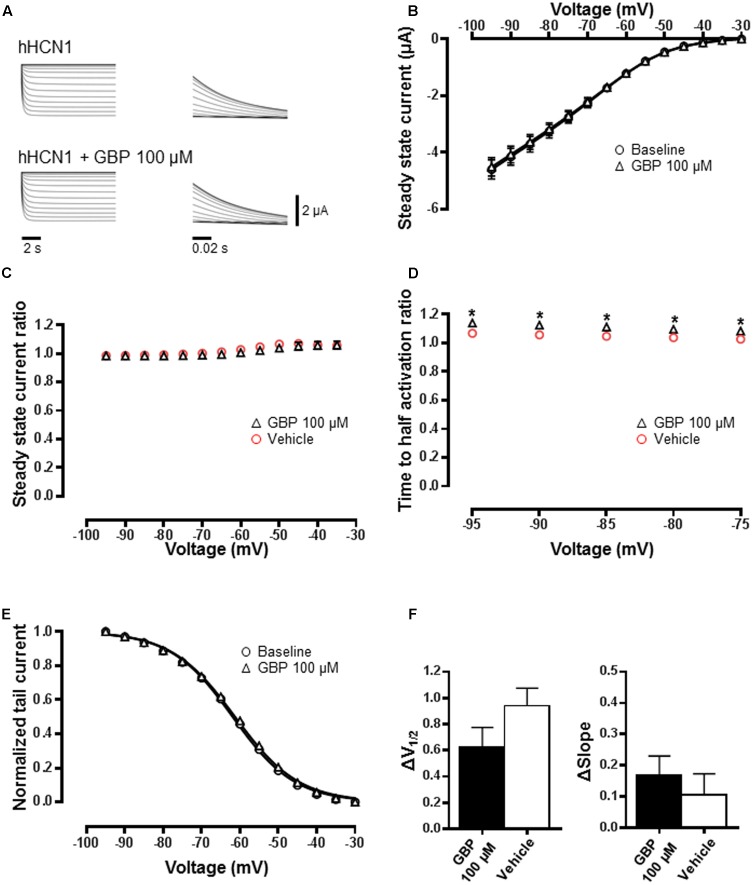
HCN1 channels are minimally affected by GBP. **(A)** Raw HCN1 channel-mediated steady-state (left) and tail currents (right) before and after 100 μM GBP. **(B)** Average steady-state current before and after GBP. **(C)** Average 100 μM GBP to vehicle steady-state current ratio at various voltages. **(D)** Average 100 μM GBP to vehicle time to half-activation ratio at various voltages. **(E)** Normalized conductance–voltage relationship constructed from tail currents. **(F)** Average shift (Δ) in the voltage of half-activation and slope of HCN1 channel by 100 μM GBP and vehicle control, relative to baseline measurements.^∗^*p* < 0.05.

Gabapentin (100 μM) had no impact on the steady-state currents of HCN1 channel-mediated current relative to baseline (**Figures 2A,B**; *n* = 30). Consistent with this, GBP (100 μM) had no effect on steady-state current across all voltages relative to vehicle control (*n* = 14) (**Figure 2C**). Gabapentin (100 μM) did slightly slow HCN1 activation kinetics across a range of voltages (**Figure 2D**; *p* < 0.05). The normalized conductance–voltage relationship of HCN1 currents was unchanged (**Figure 2E**), with no significant difference in *V*_1/2_ relative to vehicle control (**Figure 2F**; *p* = 0.2). Similarly, the slope of the HCN1 conductance–voltage relationship was unaffected (**Figure 2F**; *p* = 0.5).

**FIGURE 3:**
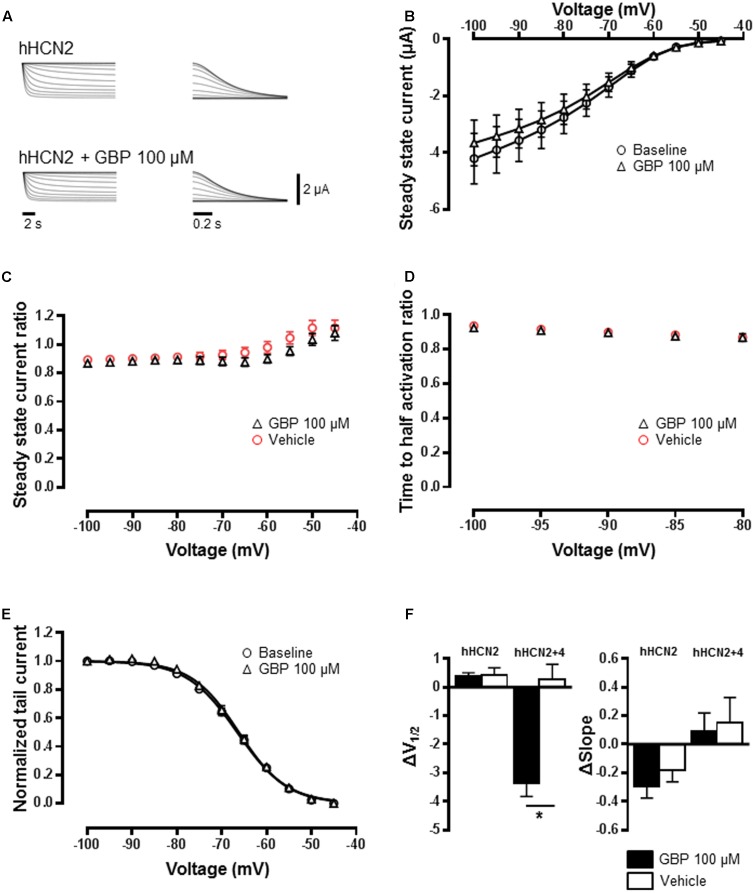
HCN2 channels are unaffected by GBP. **(A)** Raw HCN2 channel-mediated steady-state (left) and tail currents (right) before and after 100 μM GBP. **(B)** Average steady-state current before and after GBP. **(C)** Average 100 μM GBP to vehicle steady-state current ratio at various voltages. **(D)** Average 100 μM GBP to vehicle time to half-activation ratio at various voltages. **(E)** Normalized conductance–voltage relationship constructed from tail currents. **(F)** Average shift (Δ) in the voltage of half-activation and slope of HCN2 and HCN2+4 channels by 100 μM GBP and vehicle control, relative to baseline measurements.

Gabapentin had no impact on the steady-state HCN2 channel currents relative to baseline (**Figures 3A,B**; *n* = 22). Gabapentin (100 μM) also had no effect on steady-state current across all voltages relative to vehicle control (*n* = 12) (**Figure 3C**). The activation kinetics of HCN2 channels in the presence of GBP was not affected (**Figure 3D**). Normalized conductance–voltage relationship was not different from baseline (**Figure 3E**), with no significant change in *V*_1/2_ (**Figure 3F**; *p* = 0.9) or slope (**Figure 3F**; *p* = 0.4) relative to vehicle control. Given the potential for the formation of heteromeric HCN channels (Biel et al., 2009; Wahl-Schott and Biel, 2009), we tested the impact of GBP on oocytes co-injected with HCN4 and HCN2 channels. Gabapentin significantly left-shifted *V*_1/2_ of heteromeric HCN2+4 currents (*n* = 25) compared to vehicle control (*n* = 7) (**Figure 3F**; *p* < 0.05). The impact of GBP on currents mediated by HCN2+4 heteromeric channels was significantly less than that observed for HCN4 homomeric channels (HCN2+4 GBP 100 μM Δ*V*_1/2_ = -3.4 ± 0.4 mV vs. HCN4 GBP 100 μM Δ*V*_1/2_ = -5.0 ± 0.3 mV, *p* < 0.05). Interestingly, there was no effect of GBP (100 μM) on the Boltzmann slope (**Figure 3F**; *p* = 0.8).

### GBP Reduces HCN4 Channel Function in PV^+^ Inhibitory Neurons in Mouse Spinal Cord

**FIGURE 4:**
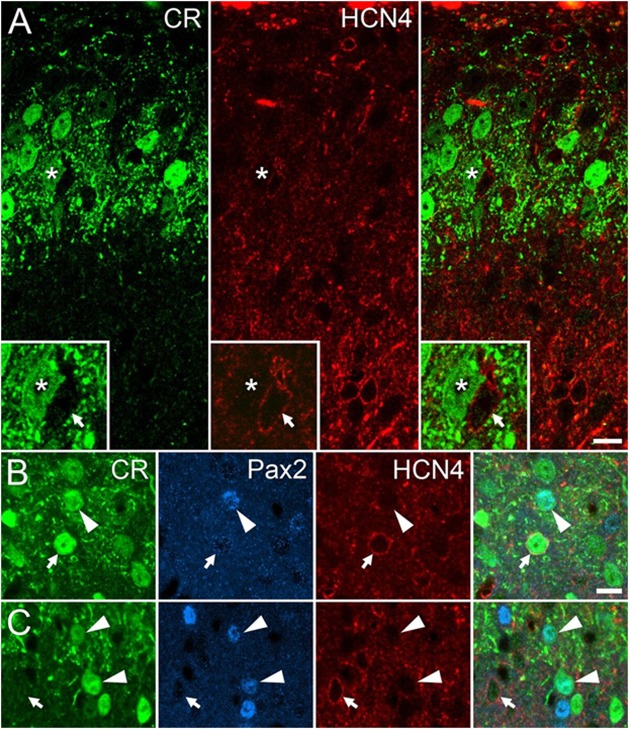
HCN4 expression in mouse dorsal horn populations. **(A)** Immunolabeling for calretinin (CR) (green) and HCN4 (red) overlaps substantially in the superficial laminae of the spinal dorsal horn, but rarely in the same cells (asterisk and inset shows the lack of HCN4 labeling in a CR neuron). (**B** and **C**) HCN4 immunolabeling is absent in Pax2-expressing (blue) CR^+^ neurons (arrowheads), however, HCN4 is detected in many neurons lacking Pax2 (arrows). Scale bar = 10 μm.

Targeted-whole-cell patch-clamp experiments have shown that PV^+^ neurons in laminae II and III of the spinal cord have a high incidence of *I*_h_ sub-threshold currents, and that ∼80% of these neurons express HCN4 channels (Hughes et al., 2012, 2013). Here, we assessed whether another population of lamina II inhibitory interneurons also known to have a high incidence of *I*_h_ currents express HCN4 channels. Approximately 15% of CR^+^ interneurons in lamina II express Pax2, an inhibitory interneuron marker, and these cells have a high incidence of *I*_h_ (Smith et al., 2015, 2016). Our immunocytochemical studies found that HCN4 immunolabeling was absent in the cell bodies of inhibitory (Pax2-expressing) CR^+^ neurons in lamina II (0 of 205 cells; 60, 66, and 79 cells analyzed per animal). Hyperpolarization-activated cation-4 immunolabeling in lamina II was seen in many cells that expressed Pax2 but lacked CR, and occasionally in CR^+^ cells that lacked Pax2, although this was not analyzed formally (**Figure 4**). As PV^+^ and CR^+^ inhibitory populations therefore appear to have distinct somatic HCN4 expression patterns, they represent good targets to assess HCN4-dependent GBP effect on *I*_h_ currents. This was assessed further in targeted patch clamp recordings made in spinal cord slices from PVeGFP or CReGFP neurons (Hughes et al., 2012; Smith et al., 2016).

**FIGURE 5:**
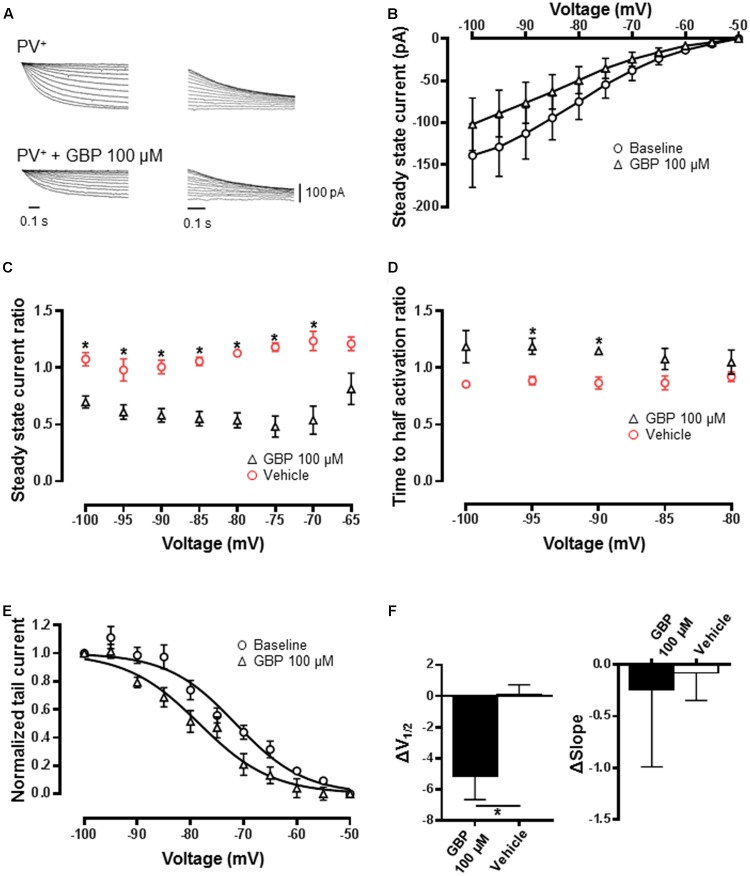
GBP reduces *I*_h_ recorded from PV^+^ inhibitory neurons. **(A)** Raw steady-state *I*_h_ (left) and tail currents (right) before and after application of 100 μM GBP recorded from PV^+^ neurons. **(B)** Average steady-state current before and after GBP recorded from PV^+^ neurons. **(C)** Average 100 μM GBP to vehicle steady-state current ratio at various voltages. **(D)** Average 100 μM GBP to vehicle time to half-activation ratio at various voltages. **(E)** Normalized conductance–voltage relationship constructed from tail currents recorded from PV^+^ neurons. **(F)** Average shift (Δ) in the voltage of half-activation and slope of PV^+^ neurons by 100 μM GBP and vehicle control, relative to baseline measurements. ^∗^*p* < 0.05.

Whole-cell patch-clamp recordings from PVeGFP neurons were used to measure *I*_h_ (**Figures 5A,B**, *n* = 10). The addition of the broad-spectrum blocker, ZD7288 (100 μM) abolished currents evoked by hyperpolarizing voltage excursions (*n* = 3, ??). Gabapentin (100 μM) significantly reduced steady-state current across all voltages except -65 mV relative to vehicle control (**Figure 5C**; *n* = 10, *p* < 0.05). There was also a significant slowing in the time to activation at -90 and -95 mV (**Figure 5D**; *p* < 0.05). Similar to heterologous expressed HCN4 channels, the normalized conductance–voltage relationship was left-shifted by ∼5 mV relative to baseline (**Figures 5E,F**). *V*_1/2_ estimated by a Boltzmann fit was significantly shifted relative to vehicle control (**Figure 5F**; *p* < 0.05). No significant difference in the slope of the curve was observed (**Figure 5F**; *p* = 0.9).

**FIGURE 6:**
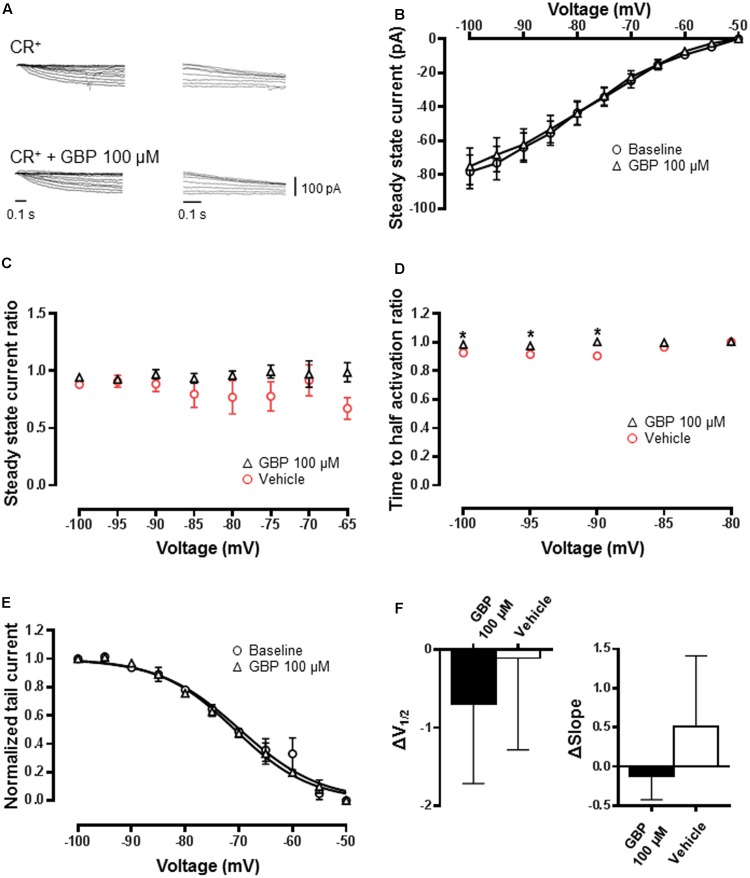
GBP minimally affects *I*_h_ recorded from CR^+^ inhibitory neurons. **(A)** Raw steady-state *I*_h_ (left) and tail currents (right) before and after application of 100 μM GBP recorded from CR^+^ neurons. **(B)** Average steady-state current before and after GBP recorded from CR^+^ neurons. **(C)** Average 100 μM GBP to vehicle steady-state current ratio at various voltages. **(D)** Average 100 μM GBP to vehicle time to half-activation ratio at various voltages. **(E)** Normalized conductance–voltage relationship constructed from tail currents of CR^+^ neurons. **(F)** Average shift (Δ) in the voltage of half-activation and slope of CR^+^ neurons by 100 μM GBP and vehicle control, relative to baseline measurements. ^∗^*p* < 0.05.

In recordings from CReGFP neurons, GBP had no impact on biophysical properties (**Figures 6A,B**; *n* = 12) including steady-state current relative to vehicle control (**Figure 6C**, *n* = 12). There was a small but significant slowing in the time to activation across at -90 to -100 mV (**Figure 6D**; *p* < 0.05). The normalized conductance–voltage relationship was unchanged (**Figure 6E**) with *V*_1/2_ and curve slope estimated by a Boltzmann fit not significantly different relative to vehicle control (**Figure 6F**; *p* = 0.7 and 0.4, respectively). Furthermore, GBP (100 μM) shifted *V*_1/2_ significantly more in PV^+^ neurons compared to CR^+^ neurons (CR^+^ GBP 100 μM Δ*V*_1/2_ = -0.7 ± 1.0 mV vs. PV^+^ GBP 100 μM Δ*V*_1/2_ = -5.2 ± 1.5 mV, *p* < 0.05). Therefore, our recordings showed that *V*_1/2_ of *I*_h_ was shifted significantly by GBP in a population that exhibits robust expression of HCN4, whereas *I*_h_ is insensitive to GBP in a population that lacks HCN4.

## Discussion and Conclusion

Hyperpolarization-activated cation channels are potential drug targets for several disease states including pain and epilepsy (Reid et al., 2012; Benarroch, 2013; DiFrancesco and DiFrancesco, 2015; Tibbs et al., 2016). The different biophysical properties and cellular distributions of the varying HCN subtypes give them unique roles within the central nervous system. Hyperpolarization-activated cation channel isoform-selective modulation is therefore likely to generate different efficacy and side-effect profiles. A major issue in the field has been the discovery of HCN isoform-selective compounds (Novella Romanelli et al., 2016). Here, we demonstrate that GBP has a robust impact on HCN4-mediated currents. The left-shift in activation voltage was observed in both heterologously expressed human channels and *I*_h_ of PV^+^ inhibitory dorsal horn neurons known to express HCN4 channels (Hughes et al., 2012, 2013). Gabapentin was essentially without effect on heterologously expressed human HCN1 and HCN2 channels and in CR^+^ inhibitory dorsal horn neurons, which exhibit *I*_h_ currents but do not express somatic HCN4 channels. To the best of our knowledge, our data provide the first evidence of a drug with almost full selectivity for the HCN4 channel subtype over other centrally expressed HCN channels. Moreover, GBP provides a unique tool to investigate the functional role of HCN4 channels in the central nervous system.

The native neuronal environment is complex, with several unknown variables that include exact HCN channel subunit composition and unknown constitutive second messenger modulation. Unlike heterologous expressed homomeric HCN4 channels, recordings from PV^+^ inhibitory dorsal horn neurons failed to show a GBP-mediated change in the Boltzmann slope. Interestingly, in expressed heteromeric HCN2+4 channels GBP did not affect the slope despite having a significant effect of on *V*_1/2_. This is consistent with the idea that *I*_h_ in PV^+^ neurons is mediated by heteromeric HCN channels (Hughes et al., 2012). It is also important to note that we have not ruled out a contribution of the HCN3 channel, although there is no evidence to suggest that this subunit is expressed in PV^+^ inhibitory dorsal horn neurons. Gabapentin subtly increased the time to half activation in CR^+^ neurons that lack HCN4 channels. This is consistent with the small increase caused by GBP in homomeric HCN1 channels, although further efforts are required to confirm the exact HCN channel expression patterns in these cells. In summary our *in vitro* and *ex vivo* results are consistent with the idea that GBP can modulate *I*_h_ in a subunit specific manner.

One important finding from this study was the apparent lack of somatic expression of HCN4 channels in the Pax2-expressing CR cells in lamina II. Although HCN channels containing HCN1 and HCN2 subunits are known to be preferentially expressed in the distal dendrites of cortical and hippocampal pyramidal cells (Notomi and Shigemoto, 2004), this restricted expression pattern is not seen for the HCN4 subunit in the same cells nor has it been described in neurons from other subcortical regions (Hughes et al., 2013). On the contrary, several examples of cells that express HCN subunits in both the brainstem and spinal cord show continuity in HCN subunit expression throughout their somatodendritic domain (Milligan et al., 2006; Hughes et al., 2013). Although we cannot dismiss the possibility of a restricted pattern of HCN4 expression confined to dendritic domains in Pax2-expressing CR using the approaches described here, we conclude that our failure to see HCN4 labeling in the cell body of these cells reflects the labeling patterns described previously for HCN subunits in the spinal cord, and is therefore consistent with an absence of HCN4 elsewhere in the cell.

Gabapentin had no impact on expressed HCN4 channel biophysical properties at 50 μM. Hundred micromolar is likely to be at the upper end of the expected plasma concentrations of patients taking high doses of the drug (Berry et al., 2003) with cerebral spinal fluid levels likely to be less (Ben-Menachem et al., 1995). This said it is not possible to estimate the concentration of GBP achieved at receptors in the central nervous system. It is therefore not unreasonable to consider HCN4 channels as a potential molecular target of GBP. Pharmacological studies have demonstrated that blocking HCN channels can have analgesic and anticonvulsant effects, for example, systemic injection of the broad-spectrum HCN blocker, ZD7288, reduces neuropathic pain behavior in rodent models (Chaplan et al., 2003; Lee et al., 2005; Luo et al., 2007). Interestingly, direct cortical infusion of ZD7288 can similarly produce analgesic effects (Cordeiro Matos et al., 2015; Koga et al., 2015). Moreover, ivabradine, a clinically used broad-spectrum blocker of HCN channels can suppress mechanical allodynia in rodent models of neuropathic pain (Noh et al., 2014; Young et al., 2014). To date there is no evidence that specifically implicates HCN4 channels in the pathogenesis of neuropathic pain. However, HCN4 channels are well placed to modulate the activity of PV^+^ neurons that have been shown to be critical modulators of mechanical hypersensitivity that develops following nerve injury (Petitjean et al., 2015).

Broad-spectrum block with ZD7288 also reduces seizure susceptibility in several animal models of epilepsy including electrically induced paroxysmal discharges (Kitayama et al., 2003), generalized seizures in Mongolian gerbils (Matsuda et al., 2008), and maximal electroshock-induced seizures (Luszczki et al., 2013). There is some associative evidence suggesting increased HCN4 channel function may be part of the mechanism underlying hyperexcitability in epilepsy. For example, HCN4 mRNA levels are increased in the pilocarpine rodent model of temporal lobe epilepsy, which correlates with an increase in *I*_h_ in dentate granule cells (Surges et al., 2012). Also, thalamocortical neurons switch from predominant HCN2 to a predominant HCN4 channel expression pattern in a cortical stroke model in which seizures developed (Paz et al., 2013). Clearly, future investigations are needed to identify the role HCN4 channels play in both seizure susceptibility and neuropathic pain, and whether the acute effects of GBP can be attributed to this molecular target.

Hyperpolarization-activated cation-4 channels are highly expressed in cardiac tissue, especially in the sinoatrial node, where they are a major driver of rhythmicity (Scicchitano et al., 2012). Given this expression pattern, along with our data, it is noteworthy that cardiovascular adverse events are rarely reported in patients taking GBP. This is consistent with the fact that even significant block of HCN4 channel function with the clinically used broad-spectrum *I*_h_ blocker, ivabradine, generally results only in a self-limiting bradycardia (Savelieva and Camm, 2008). Careful evaluation may be warranted in patients taking GBP to gauge whether the shift in HCN4 *V*_1/2_ observed here is sufficient to subtly alter cardiac function. Interestingly, a recent meta-analysis of GBP use on hemodynamic response during endotracheal intubation concluded that the drug significantly attenuated increases in heart rate and blood pressure due to the procedure (Doleman et al., 2016). This is consistent with HCN4 channel block, although it could equally reflect other physiological changes including the attenuation of the sympathetic stress response elicited by intubation (Todd et al., 2012).

Our results are not consistent with experiments completed in hippocampal slices. In contrast to the GBP-mediated reduction in *I*_h_ that we see, *I*_h_ currents are increased in both CA1 pyramidal and inhibitory interneurons (Peng et al., 2011; Surges et al., 2003). This is surprising as HCN1 and HCN2 are highly expressed in the CA1 region of the rat hippocampus (Notomi and Shigemoto, 2004) yet, GBP had minimal impact on human HCN1 or HCN2 channels expressed in oocytes suggesting that this effect may be through a secondary mechanism. This discrepancy may also be due to the absence of neuron-specific trafficking and intracellular signaling mechanisms in the oocyte expression system, although the lack of a GBP effect in *I*_h_ in CR^+^ dorsal horn neurons, which lack somatic HCN4 channels, partly compensates for this limitation. Regardless, it will be important for future work to investigate how GBP modulates *I*_h_ in regions of the brain in which HCN4 is more robustly expressed.

## Conclusion

We provide evidence that GBP can reduce human HCN4 channel function by causing a hyperpolarizing shift in the voltage of activation. This action is mostly limited to HCN4 channels, with minimal changes observed for HCN1 channels and none for HCN2 channels. Similarly, the *I*_h_ in spinal cord PV^+^ inhibitory neurons is reduced by GBP. There is a clear paucity of HCN subunit selective drugs currently available (Novella Romanelli et al., 2016). Gabapentin provides a good lead compound on which other selective compounds could be developed. Further, efforts aimed at understanding the structural–function relationship of GBP binding to HCN4 channels will help in this endeavor. Gabapentin action on HCN4 channels has the potential to contribute to analgesic and anticonvulsant activity, however, future experiments will be needed to establish a causal link between HCN4 channel block and reduced neuronal/circuit excitability within the central nervous system.

## Author Contributions

H-ST, KS, AMP, KB, ML, RH, and RR completed experimental work. IF, DH, BG, SP, and CR had intellectual input into the design and analysis of experiments performed. First draft was completed by H-ST and CR. All authors contributed to the final version.

## Conflict of Interest Statement

The authors declare that the research was conducted in the absence of any commercial or financial relationships that could be construed as a potential conflict of interest.

## Supplementary Material

FIGURE S1 ZD7288 robustly reduces *I*_h_ recorded from PV^+^ inhibitory neurons. **A**. Raw steady-state *I*_h_ **(left)** and tail currents **(right)** before and after application of 100 μM ZD7288 recorded from PV^+^ neurons. **B**. Average steady-state current before and after ZD7288 recorded from PV^+^ neurons.
