# Supplementary figures and images for: Gabapentin Modulates HCN4 Channel Voltage-Dependence
Source: Front Pharmacol. 2017 Aug 21;8:554. doi: 10.3389/fphar.2017.00554 (PMC5566583; doi:10.3389/fphar.2017.00554)

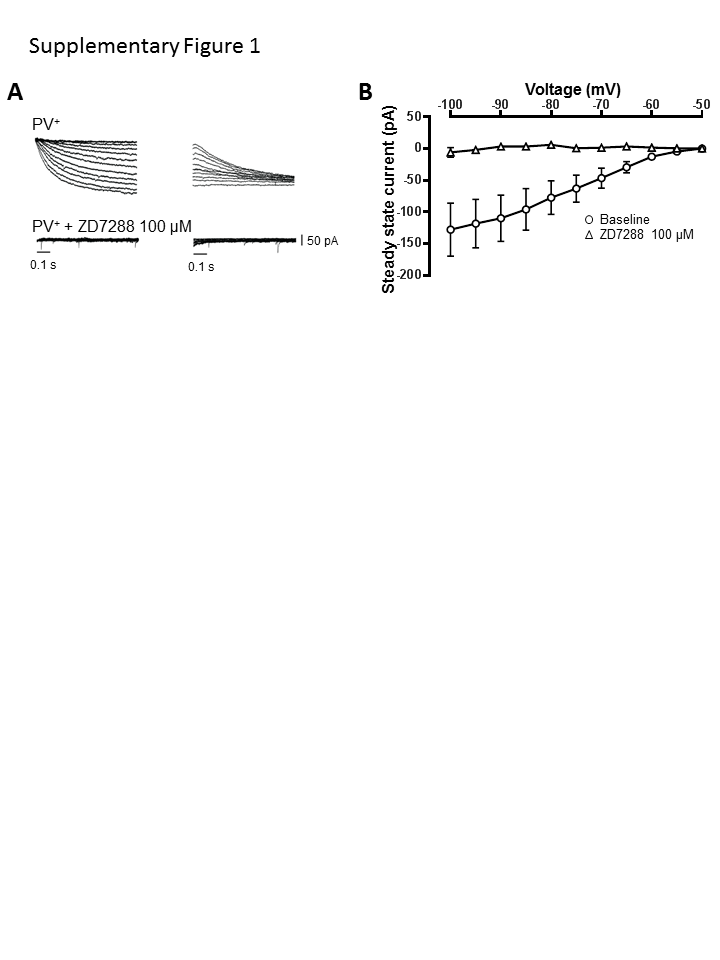

Supplement: FIGURE S1 — ZD7288 robustly reduces Ih recorded from PV+ inhibitory neurons. A. Raw steady-state Ih (left) and tail currents (right) before and after application of 100 μM ZD7288 recorded from PV+ neurons. B. Average steady-state current before and after ZD7288 recorded from PV+ neurons. [file Image_1.TIF]
